# Acoustic analysis and detection of pharyngeal fricative in cleft palate speech using correlation of signals in independent frequency bands and octave spectrum prominent peak

**DOI:** 10.1186/s12938-020-00782-3

**Published:** 2020-05-27

**Authors:** Fei He, Xiyue Wang, Heng Yin, Han Zhang, Gang Yang, Ling He

**Affiliations:** 1 College of Electrical Engineering, Sichuan University, 610065 Chengdu, China; 2 West China Hospital of Stomatology, Sichuan University, 610041 Chengdu, China

**Keywords:** Cleft palate speech, Pharyngeal fricative speech, Correlation of independent frequency bands, Energy region, Prominent peak

## Abstract

**Background:**

Pharyngeal fricative is one typical compensatory articulation error of cleft palate speech. It passively influences daily communication for people who suffer from it. The automatic detection of pharyngeal fricatives in cleft palate speech can provide information for clinical doctors and speech-language pathologists to aid in diagnosis.

**Results:**

This paper proposes two features (CSIFs: correlation of signals in independent frequency bands; OSPP: octave spectrum prominent peak) to detect pharyngeal fricative speech. CSIFs feature is proposed to detect the distribution characteristics of frequency components in pharyngeal fricative speech caused by the changed place of articulation and movement of articulators. While OSPP is presented to reflect the concentration degree of prominent peak which is closely related to the place of articulation in pharyngeal fricative, both features are investigated to relate to the altered production process of pharyngeal fricative. To evaluate the capability of these two features to detect pharyngeal fricative, we collected a speech database covering all the types of initial consonants in which pharyngeal fricatives occur. In this detection task, the classifier used to discriminate pharyngeal fricative speech and normal speech is based on ensemble learning.

**Conclusion:**

The detection accuracy obtained with CSIFs and OSPP features ranges from 83.5 to 84.5% and from 85 to 87%, respectively. When these two features are combined, the detection accuracy for pharyngeal fricative speech ranges from 88 to 89%, with an AUC (area under the receiver operating characteristic curve) value of 93%.

## Background

Cleft palate is one of the most common craniofacial alterations and birth defects [1]. It represents the third most frequently occurring congenital deformity in the world [2]. A large number of patients suffering from cleft palate are influenced by compensatory articulation error due to the existence of velopharyngeal insufficiency. Pharyngeal fricative is one typical compensatory articulation error caused by velopharyngeal insufficiency in cleft palate speech [3]. This speech disorder passively influences daily life for people who suffer from it.

Pharyngeal fricatives are produced when the base of the tongue approaches the posterior pharyngeal wall [4, 5]. This specific manner of articulation of the pharyngeal fricative is formed by constricting airflow through a narrow passage in the place of articulation. The place of articulation of the pharyngeal fricative is the pharynx, with the root of the tongue against the back of the pharynx. It operates differently from that place of articulation in normal speech. In particular, the manner of articulation may change sometimes. These changes in the production process of the pharyngeal fricative mainly influence the pronunciation of the initial consonant [3]. Sometimes, the speech production process of patients involves the nasalization [6, 7], and the pronunciation of the vowel is also influenced [8]. Thus, the quality of pharyngeal fricative speech could reduce, with a consequent reduction in the clarity of speech [9].

In clinic, three classes of approaches are generally used for the diagnosis of pharyngeal fricatives. (1) Medical instruments: clinicians use invasive instruments such as endoscopes [10, 11], lateral cephalometric radiographs on X-ray machines [3], and Opti-Speech system [12] to observe if there exist abnormal movements of articulators in the production process of speech. These methods can provide an objective diagnosis for pharyngeal fricatives. However, they are time consuming, invasive, intrusive, and costly. They also cause pain and discomfort to patients, especially young children. In addition to these invasive medical instruments, a nasal meter [13, 14] is also used to assess pharyngeal fricative by evaluating the nasal score of some specific syllables. This method gives evaluation results quickly and effectively. However, there is an unresolved debate over whether pharyngeal fricative speech has a higher nasal score [13, 14] than normal speech. (2) Subjective methods: speech-language pathologists (SLPs) listen to live or recorded speech signals and perceptually rate them. Then, they tailor treatment options for patients with pharyngeal fricative according to the evaluation result. This process of speech therapy has continued for many years. It is expensive and heavily dependent on the subjective experience of SLPs. Thus, these methods impose a heavy burden on therapists and patients. (3) Articulation evaluation system: the Ankara Articulation Test [15] and System Analysis Observation [16] are often adopted to assess the pronunciation of patients. The Ankara Articulation Test [15] scores the pronunciation of children by asking them to read given words from pictures. The score is obtained from the phoneme mistakes detected in their words. The system analysis observation [16] can provide a nonstandardized assessment of the respiration, phonation, articulation, and prosody of speech motor subsystems. These two evaluation methods are time consuming and nonstandardized. In addition, they require a large amount of manual operations.

With the aim of relieving the pain of patients with pharyngeal fricative and exploring more efficient approaches, many studies have been carried out based on speech signal processing in recent years. Speech signal processing is a noninvasive technique based on digital signal processing, which is an efficient tool for the objective diagnosis of speech disorders [17]. In the field of pharyngeal fricative speech processing, some researchers use Speech Analyzer 1.5, Universal Signal Spectrum Analysis, and CSL-4150B to study several characteristics of speech signals [18–20]. These characteristics include the speech spectrum, fundamental frequency, formants, and pitch. These instruments provide a relatively objective assessment of pharyngeal fricative for clinicians and SLPs. However, since the output information of these digital instruments requires the judgement by clinicians and SLPs, these methods can not achieve automatic diagnosis for pharyngeal fricative. In clinic, clinical doctors need more objective measurements to evaluate the speech quality of pharyngeal fricatives [21, 22]. To overcome this limitation, related studies that have realized automatic detection of pharyngeal fricative have been conducted by Xiao et al. [23, 24], He et al. [25], and Fu et al. [26]. Xiao et al. [23, 24] propose features based on one-third octave spectrum, which can reflect the characteristics of the energy distribution of speech signal. Their study focuses on the low-frequency region that is lower than 4000 Hz in the speech spectrum. However, the changes in the place of articulation and movement of articulators are closely related to high-frequency components of speech signals [27–29]. The distribution information in the high-frequency part of the speech signal is not considered in their research. Their work focuses on the initial consonant /sh/ (IPA (International Phonetic Alphabet): [ʂ]) which is more sensitive to pharyngeal fricative than other types of initial consonants. He et al. [25] present acoustic features considering only the change in the energy concentration regions of the pharyngeal fricative /s/. They propose the ICPD feature based on the central frequencies and the peak values of the energy concentration regions. There are 221 speech samples (127 pharyngeal fricatives /s/, and 94 normal speech /s/) in their classification work. Fu et al. [26] implement preliminary experiments based on speech signals collected from 10 patients and 4 control subjects. The imbalanced sample sizes of pharyngeal fricatives and normal speech could result in the overfitting of the model. In addition, their proposed features are based on the overall variations in the whole vocal tract, and do not focus on the changed place of articulation in the pharyngeal fricative. A further work to explore the biomarkers of pharyngeal fricatives is also needed.

In this paper, we aim to achieve automatic detection of pharyngeal fricative speech. The six types of initial consonants in pharyngeal fricative speech are analyzed initially. The generation of initial consonant is a dynamic process [30]. The common characteristics of these initial consonants are the change in the place of articulation and the decrease in the movement of articulators. These changes result in acoustic differentiations in pharyngeal fricative compared to normal speech [31]. In this work, two features are proposed based on these changes, namely, the correlation of signals in independent frequency bands (CSIFs) and the octave spectrum prominent peak (OSPP). CSIFs feature is proposed to establish a relation between the signals in high-energy region and the low-energy region, thereby the changes in the high- and low-frequency regions of pharyngeal fricative are emphasized. OSPP mainly focuses on the information of the dominant peak of energy-concentration region in the spectrum. The dominant peak is closely related to the place of articulation. Next, the capability of these two features is tested by speech samples with the six types of initial consonants. Compared with other studies, the major contributions of this work are summarized as follows: (1) CSIFs is proposed as a feature. It establishes the relation of the signals in high-frequency part and the low-frequency part by the self-defined high-energy region and the low-energy region in the speech spectrum of each initial consonant. This relation can reflect the distribution difference of frequency components in pharyngeal fricative speech compared with those in normal speech. (2) OSPP is proposed as a feature. The typical change in the production of pharyngeal fricative is the place of articulation. The OSPP feature is presented to represent this change by quantifying the prominent peak of the energy-concentration region. This feature is sensitive to the change in the place of articulation. (3) In this paper, the study of pharyngeal fricative covers the six types of initial consonants in which the pharyngeal fricative occurs. Analyzing all six types of initial consonants is useful for understanding the production process of pharyngeal fricatives.

## Results

This paper aims to achieve automatic detection of pharyngeal fricative speech. CSIFs and OSPP features are proposed to reflect the changes in the production process of pharyngeal fricative speech. In this section, the significance test for both proposed features and the experiments of pharyngeal fricative speech detection using these two features are conducted.

This section is composed of five parts. In the first part, the classifier used to evaluate the performance of the proposed features is described. In this classifier, several weak classifiers are trained to form a strong classifier. In the second and third part, the results of CSIFs and OSPP features calculated from normal speech and pharyngeal fricative speech are described and analyzed. In the fourth and fifth parts, the results of significance test for these two features and the detection results of pharyngeal fricative speech using the features are given and discussed.

### Bagging of ensemble learning classifier

In our research, the pharyngeal fricative speech detection task is performed by applying the bagging of the ensemble learning classifier. The idea of ensemble learning is principally based on the theoretical cornerstone that the generalization ability of an ensemble is usually much stronger than that of a single learner [32–34]. There are 30 learners in the training process of this classification model. The ensemble of the 30 learners can improve the generalization of this classifier. In addition, the classifier ensembled by the bagging method can work well for data with perturbation [35]. It has good robustness in different classification tasks to classify different data sets. The speech database in this work is composed of six types of initial consonants. The perturbations for different types of initial consonants are different. This classifier is suitable for the classification of the pharyngeal fricative speech and normal speech.

**Fig. 1:**
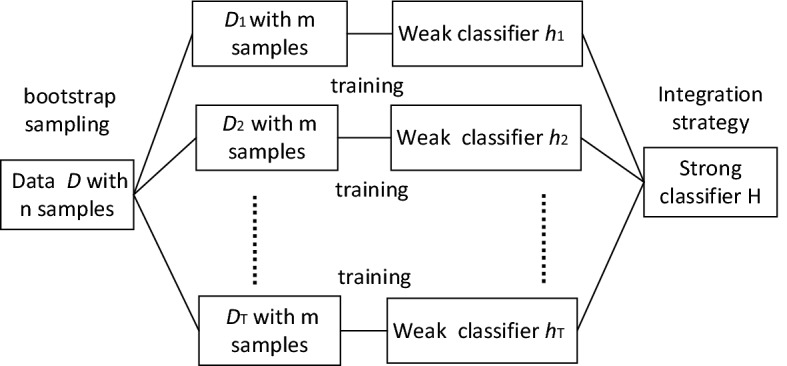
The block diagram of ensemble learning bagging

In this work, this classifier is a bootstrap-aggregated ensemble of decision trees. The decision tree algorithm includes three types of nodes, the root node, internal nodes, and end-note or target [36]. This tree algorithm uses splitting criteria to break an end-note to form a tree. This means that the root node that consists of the entire dataset is divided into subgroups by using all predictors. In this work, 30 learners of decision trees are included in the training process for this classification model. The classification performance of the model depends on the stability of the base classifiers [32]. All the base classifiers are mutually independent. They are trained using different train sets {$D_{i}$ } (*i *= 1, 2…, T), which are formed by bootstrap sampling on the original database. The final ensemble classifier H predicates the label of speech signals by majority voting of each base classifier. The output class has more votes by these base classifiers. In this work, each initial consonant is segmented into *N* frames. The proposed CSIFs and OSPP features are calculated based on the frames. Then, there are *N* or 2*N feature values that represent each initial consonant. They are input into this classifier as training parameters. This process is illustrated by Fig. 1. The weak base classifier $h_{i}$ is trained on the training data set $D_{i}$. All weak classifiers are combined to form a strong classifier.

This classifier is applied to evaluate the performance of automatic pharyngeal fricative detection system using CSIFs and OSPP features based on k-fold cross-validation (k-CV) algorithm. The k-CV algorithm is commonly used in the field of machine learning [37–41]. In the k-CV technique, the training dataset is randomly divided into k subsets with approximately equal size. In each iteration, one of the subsets is used for testing and the remaining data are used for training. The error of the subset in each iteration is calculated and the mean of the errors in all iterations gives the performance of the algorithm [37]. In this work, the sample sizes of pharyngeal fricatives and normal speech are balanced. This k-CV algorithm is suitable for the matched sample sizes in the classification task [38]. This work uses 10 instance of k-fold cross-validations.

### The experimental results and analysis of CSIFs feature

**Fig. 2:**
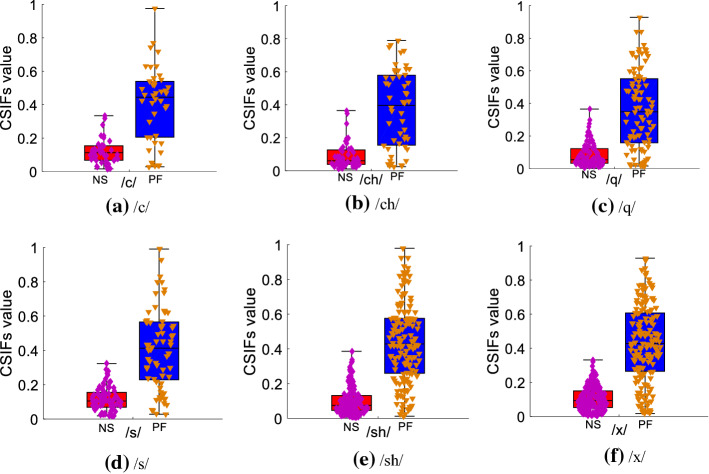
The CSIFs values for each type of initial consonant. **a** /c/; **b** /ch/; **c** /q/; **d** /s/; **e** /sh/; **f** /x/

In this subsection, the CSIFs values of the six types of initial consonants are presented to observe their distribution characteristics in normal speech and pharyngeal fricative speech. These results for each type of initial consonant are shown in Fig. 2 using boxplot. Boxplot is an exploratory data analysis tool [42] that uses a five-point (the median, upper quartile (Q3), lower quartile (Q1), lowest point, and highest point [43]) summary to reflect the distribution information of different data sets. These five parameters can show the degree of concentration and distribution range of different data sets.

In Fig. 2, PF represents the CSIFs values of pharyngeal fricative speech, while NS represents those of normal speech. The symbols of diamond and inverted triangle on the top layer of boxplot represent CSIFs values of samples. Figure 2 demonstrates that the maximum values of CSIFs for pharyngeal fricative speech are close to 1, while the CSIFs values of normal speech are all smaller than 0.4. The median, Q3, and Q1 of pharyngeal fricative speech are larger than those of normal speech. The CSIFs values of normal speech range from 0.01 to 0.397, while those of pharyngeal fricative speech range from 0.10 to 0.989. These results show that CSIFs values of pharyngeal fricative speech concentrate in a higher range than those of normal speech.

The calculation of CSIFs is based on the relation between self-defined HER and LER. The HER and LER are defined considering the amplitude distribution of frequency components in the speech spectrum of pharyngeal fricative speech and normal speech. They are closely related to the production process of speech signals. The difference in the distribution range of CSIFs values in pharyngeal fricative speech and normal speech is also caused by the different production processes. Speakers with pharyngeal fricative tend to contract pharyngeal muscle and make the posterior displacement of tongue to form a pinch point [44]. Meanwhile, the movements of articulators in the oral cavity decrease [45]. Pharyngeal fricative speech and normal speech are produced at different places of articulation with different movements of articulators. The places of articulation and the movements of articulators have great influence on the distribution of frequency components, especially components in high-frequency region [30]. In pharyngeal fricative speech, its low-frequency components and high-frequency components are both mainly produced near pharynx [23]. In normal speech, the high-frequency components are mainly produced in the oral cavity and/or lip. Thus, the HER and LER in normal speech and pharyngeal fricative speech have different distribution characteristics. Their frequency components of the LER are uniformly distributed and are similar to the distribution of pure turbulent noise [46], while the LER of pharyngeal fricative speech has a wider frequency range than that in normal speech. Meanwhile, the HER in normal speech has a wider frequency range and includes more frequency components than that in pharyngeal fricative speech. This indicates that the signals in the HER and LER of normal speech have greater differences in the amplitude variations and frequency range than those of pharyngeal fricative speech. Therefore, the CSIFs values of normal speech are distributed in a lower range than those of the pharyngeal fricative. This result is consistent with research [47], which has introduced the relation between turbulent noise and other signals.

### The experimental results and analysis of OSPP

**Fig. 3:**
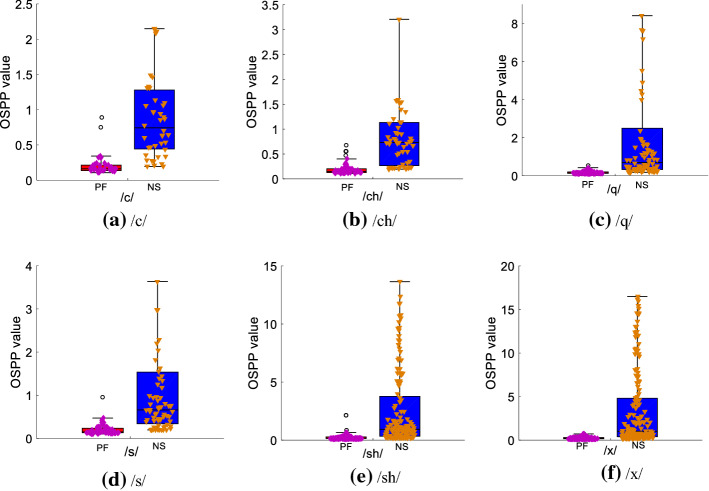
The OSPP values for each type of initial consonant. **a** /c/; **b** /ch/; **c** /q/; **d** /s/; **e** /sh/; **f** /x/

In this part, the OSPP values of the six types of initial consonants are presented to analyze the overall distribution of the prominent peaks in normal speech and pharyngeal fricative speech using the boxplot. The OSPP values of initial consonants /c/, /ch/, /q/, /s/, /sh/, and /x/ are illustrated in Fig. 3.

Figure 3 illustrates that OSPP values of pharyngeal fricative speech are with narrow distribution ranges (0–0.5) in each type of initial consonant, while OSPP values of normal speech have different distribution ranges in different types of initial consonants. The common point of OSPP values in normal speech is that OSPP values of normal speech mostly distribute in higher ranges than those of pharyngeal fricative speech.

The difference in the distribution range of OSPP values between normal speech and pharyngeal fricative speech is mainly caused by the change in the place of articulation. Speakers suffering from pharyngeal fricative are accustomed to changing the place of articulation to keep the manner of articulation [44]. Their place of articulation is in the pharynx that is formed by posterior displacement of the tongue. This place of articulation is more posterior than the correct place of articulation in the production of normal speech. This change in the place of articulation results in a larger anterior oral cavity in pharyngeal fricative than that in normal speech. Therefore, the energy-concentration regions of speech tend to shift to the lower frequency part, and the degree of energy concentration in higher frequency part decreases [23, 24]. The OSPP feature aims to evaluate the concentration degree of the prominent peak of the energy-concentration region in the speech signal. It can reflect the change in the energy-concentration region caused by different places of articulation. In our research, OSPP values of normal speech distribute in a higher range than those of pharyngeal fricative speech.

### Significance test of the proposed features CSIFs and OSPP

Significance testing is a common technique in statistics to evaluate the significant difference in several data sets [48]. It has been widely utilized to evaluate the discrimination capabilities of different feature sets in the classification tasks [49–51]. In our work, for each proposed feature, the significance test is adopted to estimate its capability for discriminating pharyngeal fricative speech and normal speech. This significance test is carried out based on these hypotheses as follows.

H = 1: There are significant differences in this feature between pharyngeal fricative speech and normal speech
H = 0: There are no significant differences in this feature between pharyngeal fricative speech and normal speech

There are six types of initial consonants in our research. The significant test results are listed in Table 1, including the significance level, the *p* value (probability of test error occurring), and the result of the hypothesis.

**Table 1:** The significance test results of the proposed features

Feature	Initial consonant	Significance level (%)	*p*	Hypothesis
CSIFs	/c/	< 1	2.85e−12	1
	/ch/	< 2	2.91e−12	1
	/q/	< 1	1.07e−23	1
	/s/	< 1	1.51e−17	1
	/sh/	< 1	1.08e−45	1
	/x/	< 1	1.44e−60	1
OSPP	/c/	< 1	5.13e−07	1
	/ch/	< 1	0.011	1
	/q/	< 1	6.79e−06	1
	/s/	< 1	4.41e−04	1
	/sh/	< 1	4.77e−08	1
	/x/	< 1	2.37e−10	1

As shown in Table 1, the significance differences based on the two proposed features for each type of initial consonant are presented. The significance level of CSIFs and OSPP features is less than 1%, except for CSIFs values in the initial consonant /ch/. Furthermore, the *p*-value is much less than 0.01. These parameters indicate that CSIFs and OSPP features have significant differences between pharyngeal fricative speech and normal speech for the six types of initial consonants.

### Automatic pharyngeal fricative speech detection results using OSPP and CSIFs

In this research, CSIFs and OSPP are proposed to detect pharyngeal fricative in cleft palate speech. In this subsection, the results of automatic pharyngeal fricative speech detection using CSIFs and OSPP features are presented and discussed. The performances of these two features are evaluated by the ensemble learning classifier. The general performances of these two proposed features are discussed in terms of accuracy, sensitivity, specificity, and AUC. Sensitivity reflects the probability of missed diagnosis in clinical, while specificity reflects the probability of misdiagnosis. AUC is the area under the receiver operations characteristic curve. It is given to show the results more compactly [52]. It can also evaluate the classifiers on both balanced and imbalanced class distributions [53].

In this experiment, CSIFs and OSPP features are used to form different feature sets to discriminate pharyngeal fricative speech and normal speech. The experimental results of automatic detection of pharyngeal fricative speech are listed in Table 2.

**Table 2:** The results obtained with features CSIFs, OSPP, and feature set CSIFs + OSPP

Feature	Accuracy %	Specificity %	Sensitivity %	AUC
CSIFs	84 ± 0.5	77 ± 0.4	91.5 ± 1.5	90
OSPP	86 ± 1	80 ± 0.5	92 ± 1	91
CSIFs + OSPP	88.5 ± 0.5	83 ± 0.5	93.5 ± 1	93

As shown in Table 2, the detection accuracies of pharyngeal fricative using CSIFs feature range from 83.5 to 84.5%. Its sensitivity and specificity are larger than 76.6% and 90%, respectively. The detection accuracies of pharyngeal fricative using OSPP feature range from 85 to 87%. Its sensitivity and specificity are larger than 79.5% and 91%, respectively. When CSIFs and OSPP features are combined, the detection accuracies of pharyngeal fricative range from 88 to 89% (AUC equals 93%), which are larger than those results obtained from a single feature.

In pharyngeal fricative speech, the movements of articulators and the places of articulation are different from those in the production process of normal speech. Since the movements of articulators in the oral cavity of the pharyngeal fricative decrease and the places of articulation change, it is aware that the speech spectrum of pharyngeal fricative speech is modified in a wider spectral range with lower amplitudes. The main objective for CSIFs is to reflect this modification. In the calculation of CSIFs, HER and LER are defined in each initial consonant, considering the modified spectral range in pharyngeal fricative speech. Then, speech signals in the HER and LER are extracted. CSIFs can reflect this distribution difference of frequency components between pharyngeal fricative speech and normal speech by evaluating the relation of signals in HER and LER. For the OSPP feature, the prominent peak of the energy-concentration region is mainly influenced by the place of articulation. Since the posterior displacement of the tongue occurs in the production of pharyngeal fricative, its prominent peak may shift to a lower frequency part than normal speech, and the amplitude of the peak decreases. The OSPP feature focuses on characterizing the most prominent peak in the speech spectrum. The results of CSIFs and OSPP features indicate that they are useful to represent the changes occurring in the pharyngeal fricative speech. The proposal of these two features is based on the change in the pronunciation process of pharyngeal fricative. When OSPP and CSIFs features are combined to detect pharyngeal fricative, the detection accuracy increases. The discrimination between pharyngeal fricative speech and normal speech is improved. This improvement is probably caused by the combination of these two features. The information on the changes in place of articulation and movement of articulators is included in the same feature set.

To observe the classification performances of pharyngeal fricative detection on different types of initial consonants, the detection experiments of pharyngeal fricatives on each type of initial consonant are conducted. The experimental results are listed in Table 3.

**Table 3:** The detection results on different types of initial consonants based on different features

Features	Results	Type of initial consonants					
		/c/	/ch/	/q/	/s/	/sh/	/x/
CSIFs	Accuracy %	84 ± 1.5	81 ± 1	80 ± 1	79.5 ± 1	85 ± 1	85.5 ± 1.5
	Specificity %	77 ± 2	75 ± 2	71 ± 1	80 ± 4	82	79 ± 1
	Sensitivity %	91 ± 2	87 ± 2	88 ± 2	85 ± 2	90 ± 1	93 ± 1
	AUC	91 ± 1	86	84 ± 1	87	93	93
OSPP	Accuracy %	83.5 ± 2.5	84 ± 1	83.5 ± 0.5	86 ± 1	85.5 ± 0.5	85 ± 1
	Specificity %	78	77 ± 3	78	84 ± 1	83 ± 1	81 ± 1
	Sensitivity %	89 ± 2	90.0 ± 1.5	90 ± 2	88	88 ± 2	91
	AUC	92	90	90	90	89	92
CSIFs + OSPP	Accuracy %	88.5 ± 1	86.5 ± 2	85.5 ± 1	84	89 ± 1	89 ± 0.5
	Specificity %	84 ± 2	80 ± 1	78 ± 2	79 ± 1	86 ± 2	82 ± 1
	Sensitivity %	94 ± 1	92 ± 2	93 ± 1	88	93 ± 1	95 ± 2
	AUC	92	90 ± 1	90	88	94	95

As listed in Table 3, the detection accuracies of pharyngeal fricatives on different types of initial consonants using CSIFs feature range from 78.5 to 87%. The detection accuracies of pharyngeal fricatives on different types of initial consonant using CSIFs feature range from 84 to 90%. When OSPP and CSIFs features are combined, it illustrates that the accuracy, specificity, and sensitivity of pharyngeal fricative detection on different types of initial consonants are improved.

## Discussion

**Fig. 4:**
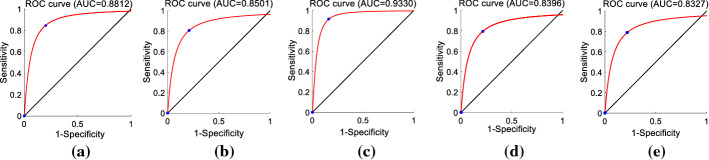
ROC curves obtained with EMAs [23], FMAs [24] and CSIFs + OSPP tested on our speech database. **a** EMAs [23] (AUC = 88%); **b** FMAs [24] (AUC = 85%); **c** CSIFs + OSPP (AUC = 93%); **d** ICPD [25] (AUC = 84%); **e** VTG + VTA + PP [26] (AUC = 83%)

In this subsection, the comparative experiments are conducted based on features proposed in previous researches [23–26] and this work. The researches [23, 24] study the spectral characteristics of pharyngeal fricative speech based on one-third octave spectrum. In research [23], the amplitudes of eight one-third octave bands form feature (EMAs) for each fricative /sh/. In research [24], different groups of amplitudes in EMAs are input into a classifier to explore the optimum feature set that is with a small standard deviation of accuracy. The optimum feature set in that study consisted of four amplitudes (FMAs) of EMAs. In research [25], He et al. present the ICPD feature based on the variation of energy in speech signals caused by the changed place of articulation in pharyngeal fricative. Fu et al. [26] extract the VTG + VTA + PP feature set from the glottal waveform and the coefficients of the vocal tract model. The capabilities of these features in detecting pharyngeal fricatives are tested using the database in this work, which contains six types of initial consonants. The test results are presented in Table 4 and Fig. 4.

**Table 4:** The results obtained using EMAs [23] and FMAs [24] feature sets

Features	Accuracy %	Specificity %	Sensitivity %	AUC
EMAs [23]	80 ± 1	74.5 ± 1	85.5 ± 0.8	88
FMAs [24]	77 ± 1	75.5 ± 1	79.5 ± 1	85
ICPD [25]	76.8 ± 0.2	74	79 ± 1	84
VTG + VTA + PP [26]	78.5 ± 0.5	79 ± 3	78 ± 2	83 ± 3

As shown in Table 4, the detection accuracies of existing features are lower than the proposed CSIFs and OSPP features (84.5%, 87%) in this work. The highest detection accuracy of pharyngeal fricative speech in this work is 89%. Moreover, the specificity, sensitivity, and AUC values of CSIFs and OSPP features are larger than those obtained with EMAs [23], FMAs [24], ICPD [25], and VTG + VTA + PP [26].

As shown in Fig. 4, the ROC curve of the CSIFs + OSPP feature set illustrates that this feature set has a balanced capability to discriminate pharyngeal fricative speech and normal speech. The AUC value of the CSIFs + OSPP feature set is approximately 93%, while the EMAs [23], FMAs [24], ICPD [25], and VTG + VTA + PP [26] are 88%, 85%, 84, and 83%, respectively.

Compared with the work of Xiao et al. [23, 24] and He et al. [25], the EMAs [23], FMAs [24], and ICPD [25] features are presented by analyzing the energy variations of the pharyngeal fricative. The proposed EMAs [23] and FMAs [24] only consider the energy variations of the initial consonant /sh/, while the proposed ICPD [25] is based on the analysis of the initial consonant /s/. However, different types of initial consonants have different characteristics of the energy variations; therefore, the three features might not perform well for each type of initial consonants. In this work, the two proposed features consider all types of initial consonants in which pharyngeal fricatives might occur.

Compared with the work of Fu et al. [26], PP [26] is the pitch period of the glottal waveform. VTA and VTG [26] are calculated from the estimated vocal tract model coefficient. The proposal of the three features is based on the overall variations of the vocal tract in the production process, and do not focus on the changed place of articulation in pharyngeal fricative. They might fail to establish a close relationship to the typical change in the place of articulation in the pharyngeal fricatives. In this work, the two proposed features CSIFs and OSPP focus on the changed place of articulation in the production process of the pharyngeal fricative speech. They perform better in detecting pharyngeal fricative speech than these features in previous work.

These features in previous work [23–26] are tested using a speech database with a limited sample size. In Xiao et al.’s work [23, 24], 161 speech samples (83 pharyngeal fricatives /sh/, 78 normal speech /sh/) are employed. In He et al.’s work, there are 221 speech samples (127 pharyngeal fricatives /s/, 94 normal speech /s/) in the classification task. In Fu et al.’s work, speech samples are composed of 297 pharyngeal fricatives and 99 normal fricatives. In this work, the two proposed features are tested with a larger speech database. This speech database covers all the types of initial consonants in which pharyngeal fricatives might occur. It consists of 1208 speech samples collected from 50 patients with pharyngeal fricative and 50 normal subjects.

## Conclusion

In our work, the main objective is to achieve automatic detection of pharyngeal fricative speech. CSIFs and OSPP features are primarily proposed based on the production process of pharyngeal fricative in cleft palate speech. Both features are useful for analyzing the place of articulation and movement of articulators in the generation process of speech signals. In this paper, their capabilities to discriminate pharyngeal fricative speech and normal speech are tested and discussed. This test is conducted on collected speech database, which consists of 1208 speech samples including six types of initial consonants. Moreover, the comparison experiments with previous studies are also conducted.

The proposed CSIFs feature aims to reflect the distribution differences of frequency components between pharyngeal fricative speech and normal speech. Since pharyngeal fricative speech has more posterior place of articulation and fewer movements of articulators in the oral cavity, its energy tends to shift to a lower frequency part than that of normal speech. Correspondingly, its low-energy region has a wider frequency range. CSIFs feature detects this distribution characteristic by evaluating the correlation of signals in high-energy region and low-energy region defined in this work. The experimental results of CSIFs note that CSIFs values calculated from normal speech are mostly lower than those calculated from pharyngeal fricative speech. The detection accuracies of pharyngeal fricative speech using CSIFs feature are above 83.5%. These results indicate that it has the capability to discriminate pharyngeal fricative speech and normal speech. In addition, the matching filter is designed to locate the high-energy region and low-energy region of each initial consonant in the calculation process of CSIFs.

In pharyngeal fricative speech, the posterior displacement of the tongue occurs in its generation process. This posterior displacement influences the energy-concentration regions in the spectrum. Generally, it results in lower amplitudes and center frequencies in the energy-concentration regions of pharyngeal fricative speech. The OSPP feature detects the change in the place of articulation in pharyngeal fricative speech by evaluating the most prominent peak of the energy-concentration regions in the spectrum. The detection accuracies of pharyngeal fricative speech obtained with OSPP feature range from 85 to 87%. These results indicate that OSPP feature has good capability to detect pharyngeal fricative speech. When the OSPP feature and CSIFs feature are combined in the same feature set, the best result is obtained with an accuracy of 89%. This result notes that the combination of OSPP and CSIFs features can improve the capability to discriminate pharyngeal fricative speech and normal speech.

A comparison with related researches on pharyngeal fricative speech detection is also conducted in this paper. Xiao Y et al. [23, 24], He et al. [25], and Fu et al. [26] have conducted preliminary experiments to detect pharyngeal fricative. Their detection results are obtained from limited speech samples. Their proposed acoustic features are tested by our database (1208 speech samples) with a detection accuracy of approximately 80%. The detection accuracy of the proposed features in our research is 89%.

Finally, the proposed features could be adopted in other speech diseases and other languages, since there exist many similarities in the place of articulation and the place of manner between Chinese and other languages. A new choice is provided for the doctors and patients. In any choice, an informed selection of the techniques that are applied to model the speech signal could help the speech therapist and the clinician to make more accurate decisions regarding the pharyngeal fricative and the treatment prescription for the patients.

## Methods

This section describes the details of the material and the proposed methods. In the first part, the details of the participants and collected speech samples are introduced. In the second part, the pronunciation process of pharyngeal fricative is described. In the following two parts, all processes of the proposed features to discriminate pharyngeal fricative speech and normal speech are clearly described, including their proposals, objectives, and detailed calculation steps.

### Pharyngeal fricative speech database

Fifty patients (32 females and 18 males, mean (± SD) age: 20.3 ± 8.1 years) with pharyngeal fricative and 50 healthy controls (29 females and 21 males, mean (± SD) age: 19.2 ± 7.8 years) participated in this research. All speech recordings are collected from the West China Hospital of Stomatology, Sichuan University. It is a prestigious and well-known medical center in China and it has the largest number of cleft palate patients in the world. This study is approved by the West China Hospital of Stomatology Institutional Review Board (WCHSIRB-CT-2013-011). All participants sign their informed consent prior to their inclusion in this research.

In the research on pharyngeal fricative speech detection, one bottleneck is the collection of speech samples. The sample size depends on the types of collected initial consonants and the amount of enrolled patients. Considering the types of initial consonants, there are six types of initial consonants (/c/, /ch/, /q/, /s/, /sh/, /x/) in which pharyngeal fricative may occur. However, in previous studies [23–26], only two types of initial consonants (/sh/ [23, 24] and /s/ [25]) are studied. Research [26] investigates the characteristics of pharyngeal fricative based on limited speech samples collected from 4 patients. In this paper, the collected speech samples contain the six types of initial consonants. These initial consonants are collected according to a specific vocabulary list designed by experienced SLPs [1]. There are 24 Chinese words in this specific vocabulary. All the collected words are frequently used in the natural communication environment. The initial consonants are segmented from these collected words using algorithm in [54]. The dataset in this work contains 1208 initial consonants (604 pharyngeal fricative speech samples, 604 normal speech samples) that are spoken by 50 patients with pharyngeal fricative and 50 normal controls.

These speech samples are collected in a specific studio and sampled with 44.1 kHz sampling rate at a resolution of 16 bits in the hospital. These speech recordings are independently rated by three SLPs. Each recording is selected for our database only if all three speech-language pathologists give the same assessment of it.

### The pronunciation process of pharyngeal fricatives

The production of speech is a complex physiological process. This physiological process culminates in the movement of articulators to control the airflow. In the production process of normal speech, the air flows from the lungs and passes through the glottis, while if the airflow is impeded by glottis adduction, a voiced sound is produced. Next, airflow enters the upper vocal tract, where articulators interact to control the airflow [3]. With the interaction of articulators, different resonant cavities are formed to generate an intelligible speech signal. This pronunciation process can be simply described as: the airflow is restricted, and these restrictions are broken through by the airflow. If there are any abnormal changes in this process, a speech signal may be generated with reduced quality and clarity, and its acoustic features could change [3, 30, 55].

**Fig. 5:**
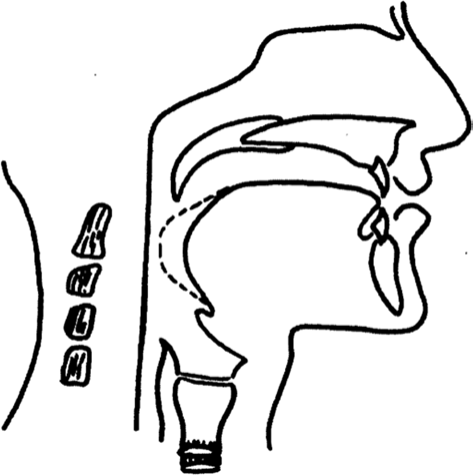
Lateral-view schematic illustration of pharyngeal fricative compensatory articulation

Pharyngeal fricative, a typical compensatory articulation error in cleft palate speech, is caused by abnormal pronunciation habits. These habits arise to compensate for velopharyngeal insufficiency [56]. Due to the velopharyngeal insufficiency, speakers with pharyngeal fricative restrict airflow in the pharynx by posterior displacement of the tongue, as shown in Fig. 5 (a lateral-view schematic illustration of the pharyngeal fricative compensatory articulation with broken line showing lingual configuration and placement [24, 57]). Thus they can generate speech with weak intraoral pressure [3]. In this process, the place of articulation changes, and the movements of articulators in the oral cavity decrease [31]. Therefore, pharyngeal fricative speech and normal speech are acoustically differentiated. The objective of this work is to explore speech features to represent these acoustic differentiations between pharyngeal fricative speech and normal speech.

### Proposed CSIFs feature based on the distribution characteristics of frequency components

Speech signal generation is a complex nonlinear process with the movements of articulators. In this process, if airflow in the lungs goes through a narrow passage in the vocal tract, turbulent noise may be generated. The generation of turbulent noise depends on the Reynolds number, which relies heavily on the size of the vocal tract and the velocity of the airflow [30, 46]. The Reynolds number is calculated by (1):

1 $$\text{Re} = {{\rho uD} \mathord{\left/ {\vphantom {{\rho uD} \mu }} \right. \kern-0pt} \mu },$$

where Re denotes Reynolds number; *ρ* and *u* are the density and speed of the airflow, respectively; $\mu$ represents the viscosity coefficient between the vocal tract and airflow, and *D* represents the size of vocal tract. The size of the human vocal tract is different for various places and manners of articulation in different pronunciations. When the Reynolds number in the place of articulation is larger than 2100, turbulent noise is generated [30].

Speakers suffering from pharyngeal fricative attempt to change their place of articulation to compensate for velopharyngeal insufficiency. The changed place of articulation is the pharynx, with the root of the tongue against the back of the pharynx. In this place of articulation, the gap between the pharynx and tongue root decreases, and the velocity of airflow increases. According to (1), the Reynolds number changes correspondingly. This change in the Reynolds number will influence the characteristics of the generated speech signal.

**Fig. 6:**
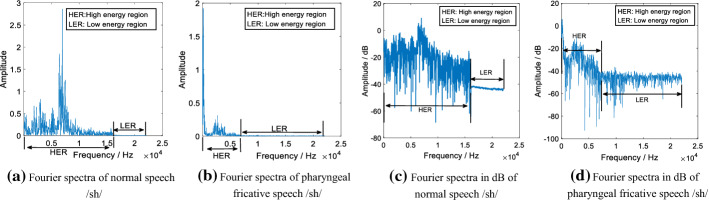
The Fourier spectra (FFT size = 1024) and the Fourier spectra in dB of initial consonant /sh/. **a** The Fourier spectra of normal speech /sh/ after FFT; **b** the Fourier spectra of pharyngeal fricative /sh/ after FFT; **c** the Fourier spectra in dB of normal speech /sh/ after FFT; **d** the Fourier spectra in dB of pharyngeal speech /sh/ after FFT

The change in the place of articulation in the production process of pharyngeal fricative results in reduced quality and intelligibility of generated speech signals. Speech quality and intelligibility are closely related to the amplitude variations of frequency components and their distribution. [30]. These information on frequency components can be reflected by the Fourier spectrum. To illustrate these differences in the Fourier spectrum between pharyngeal fricative speech and normal speech, an example is provided to show the spectra of normal initial consonant /sh/ and the pharyngeal fricative /sh/ in Fig. 6.

As shown in Fig. 6, the Fourier spectra and the Fourier spectra in dB of initial consonant /sh/ are divided into two parts according to the proposed definition method: the high-energy region (HER) and the low-energy region (LER). This definition method is based on the distribution characteristics of frequency components in normal speech and pharyngeal fricative speech. The HER is defined as the continuous frequency components with high amplitudes. The LER is defined as all frequency components in the Fourier spectrum except those in HER. In Fig. 6, the (a) and (b) are given to show the overall energy variations of normal speech and pharyngeal fricative speech /sh/, while (c) and (d) are provided to highlight the detailed amplitude variations in LER. Several typical characteristics in the defined HER and LER are summarized in the following points: (1) normal speech has richer information in the HER than pharyngeal fricative speech has. The HER of normal speech has a wider frequency range and includes more frequency components compared with that of pharyngeal fricative speech. (2) Whether speech is pharyngeal fricative speech or normal speech, its frequency components in LER have less variation than those in HER. (3) The distribution differences of the amplitudes between HER and LER in normal speech are greater than those in pharyngeal fricative speech.

**Fig. 7:**
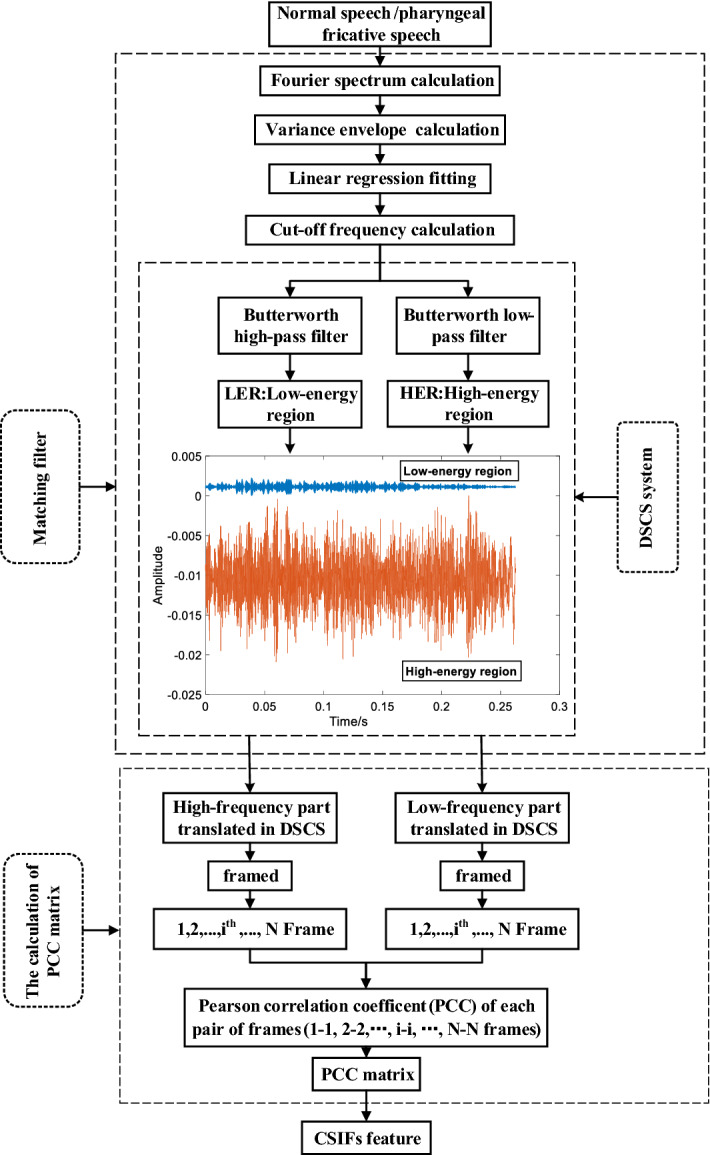
The block diagram of CSIFs calculation. The design of matching filter and the calculation of PCC matrix are two key parts in the calculation of CSIFs feature

Speakers with pharyngeal fricative restrict airflow in the pharynx by posterior displacement of the tongue [3]. This posterior place of articulation causes the energy of the pharyngeal fricative speech to shift to a lower frequency region [30]. The initial consonants in normal speech are produced in an anterior place of articulation, such as the oral cavity and/or lip. This anterior place of articulation causes the HER of normal speech to have a wider frequency range. As shown in Fig. 6, the HER of the normal speech has wider frequency range than the HER in pharyngeal fricative speech. In addition, the LER in normal speech has significantly lower amplitudes compared with its HER, as shown in Fig. 6c, while this situation is less prominent in pharyngeal fricative, as shown in Fig. 6d. The amplitude distribution of HER and LER in normal speech has greater differences than that of the pharyngeal fricative. In this work, a feature is proposed to establish a relationship between the signals in HER and LER. The block diagram of the calculation process of the proposed feature is shown in Fig. 7.

As shown in Fig. 7, the CSIFs feature is proposed based on the definition of the HER and LER. Its calculation is based on the designed matching filter and DSCS (double signal coordinate system). This calculation process is summarized as follows. First, the HER and LER of each initial consonant are separated by the designed matching filter. Second, simultaneous display of the HER and LER speech signals is realized in DSCS by coordinate transformation. Finally, the signals of HER and LER in DSCS are adopted to calculate CSIFs. The detailed technique information of CSIFs calculation is introduced in next two subsections.

### Matching filter design based on the amplitude variations of frequency components

In this work, the cut-off frequency of HER and LER is calculated based on the amplitude variations in the Fourier spectrum. However, the amplitude variations in the spectrum of each initial consonant differ in terms of various characteristics, such as vocal tract length, gender, age, place of articulation, and manner of articulation. Thus, the cut-off frequency of each initial consonant is different. To calculate the cut-off frequency, a matching filter is designed to realize self-adaptive calculation for each type of initial consonant.

This calculation process of the designed matching filter can be summarized as follows. First, the Fourier spectrum of each initial consonant is calculated and segmented into short segments. Second, the variance envelope is computed from the segmented spectrum. Then, linear regression fitting is utilized to locate the cut-off frequency of HER and LER. The detailed calculation process of cut-off frequency is described as follows:

#### Fourier spectrum calculation

The Fourier spectrum of each initial consonant is calculated by fast Fourier transform (FFT size = 1024). Then, the spectrum is segmented into short segments using the Hamming window. The selection of the window size depends on the sampling frequency of speech signals. In this work, the sampling frequency of speech signal is 44,100 Hz. A window size is selected as 689 Hz, while the window shift is 344 Hz.

#### Variance envelope calculation

**Fig. 8:**
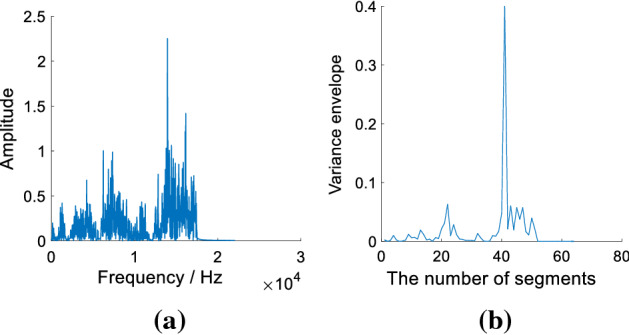
**a** The spectrum of normal initial consonant /c/; **b** the variance envelope of normal initial consonant /c/

Variance envelope $M_{v}$ (*v *= 1, 2…, *N*, where *N* is the number of segments) is composed of variance values that are calculated from spectrum segments. For each spectrum segment, its variance is calculated by (2):

2 $$M = \frac{{\sum \left( {S_{i} - \mu } \right)^{2} }}{L},$$

where $s_{i}$ (*i *= 1, 2…, *L*, where *L* is the number of data points in each spectrum segment) is the *i*th data point in the current spectrum segment. *M* represents the variance of the current spectrum segment, $\mu$ denotes the mean value of $s_{i}$. The Fourier spectrum and its variance envelope of initial consonant /c/ (IPA: [tsʰ]) are shown in Fig. 8.

As shown in Fig. 8, the Fourier spectrum is represented by the variance envelope to reflect its overall amplitude variations. In this work, the amplitudes of the defined LER are small and have low fluctuations. The variance can evaluate the fluctuation of a data set [58]; thus, the variance envelope can facilitate the calculation of cut-off frequency in each initial consonant.

#### Linear regression fitting

In variance envelope $M_{v}$, its *c*th variance $M_{c}$ (*c* = 1, 2…, *N*, where *N* is the length of variance envelope) to the last variance $M_{N}$ are fitted using linear regression. Since the LER of each initial consonant is represented by small variance values in $M_{v}$, these small variance values in consecutive segments can be reflected by small slope values of linear regression lines. Therefore, these slope values are used to locate the cut-off frequency for the HER and LER. This linear regression fitting [59] process in variance envelope is described in Algorithm 1.
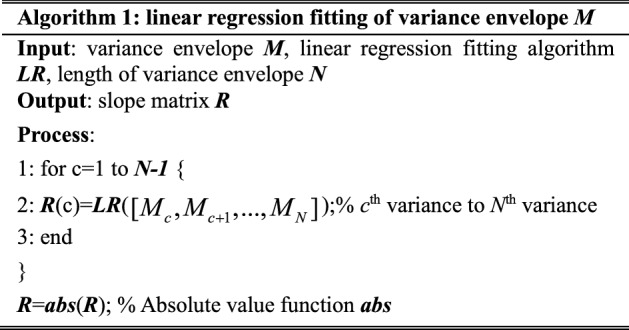


**Fig. 9:**
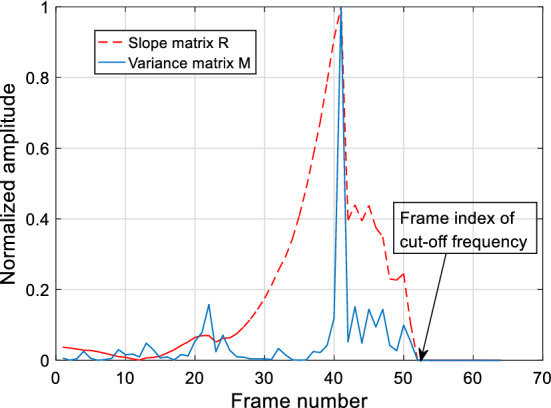
The variance envelope *M* and slopes *R* of normal fricative /c/

After the processing of the linear regression fitting algorithm in Algorithm 1, the slope matrix that contains all the slope values of the fitted linear regression lines is calculated. The normalized variance envelope and slope values are shown in Fig. 9.

As shown in Fig. 9, the dotted line is the values in slope matrix *R*, while the solid line is the variance envelope. The slope matrix *R* magnifies the difference between the HER and LER in the speech spectrum of the initial consonants.

#### Cut-off frequency calculation

**Fig. 10:**
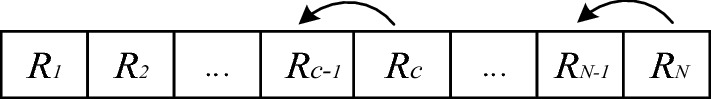
The diagrammatic sketch of pairwise comparison for slope values from back to front

In slope matrix *R*, slope value $R_{c}$ (*c *= 4, 5…, *N*-2, where *N* is the length of matrix R) is compared with $R_{c - 1}$ to locate the cut-off frequency. This comparison process is shown in Fig. 10.

**Fig. 11:**
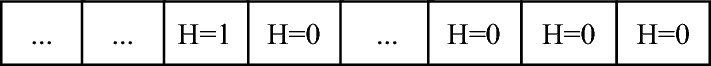
The diagrammatic sketch of the significant test results

A method is presented in the comparison process shown in Fig. 10. This method is described in Algorithm 2, in which *c_f* is the calculated cut-off frequency. *Coor* is composed of all frequency index of all spectrum segments. H denotes the results (0 or 1) of the significant test [60]. When H equals to 1, it illustrates that the compared two groups exist significant difference. The results of H in Algorithm 2 are illustrated in Fig. 11.

In steps 5–7, when H, slope $R_{c - 1}$, and $R_{c + 1}$ satisfy step 5, *c_f* is obtained by calculating the mean value of frequencies in *c*^*th*^ spectrum segment. The *c_f* is the cut-off frequency between LER and HER region.
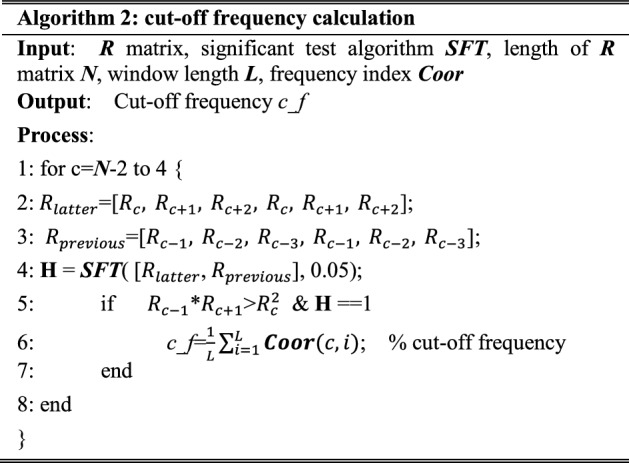


#### DSCS system

The cut-off frequency calculated above is adopted in Butterworth high-pass and low-pass filters. Thus, the HER and LER of an initial consonant are obtained. The order of these two Butterworth filters is set as 10. This order is suitable for the filtering of speech signal [61]. To have a more intuitive comparison of signals in HER and LER, a DSCS system is proposed to realize simultaneous display of them in the same coordinate axis. This simultaneous display of them is realized by coordinate transformation, which is described by (3) and (4):

3 $$NX_{H} = X_{H} - {\text{Trans}}_{H} ,$$

4 $$NX_{L} = X_{L} - {\text{Trans}}_{L} .$$

**Fig. 12:**
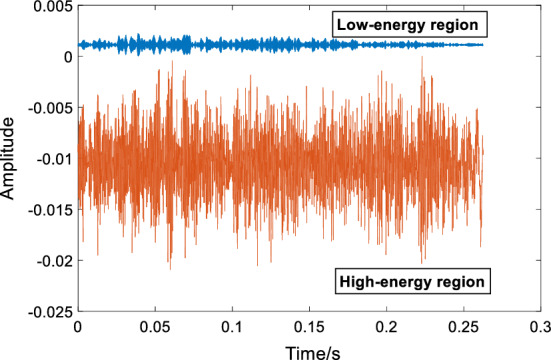
Coordinate transformation for high-frequency part and low-frequency part in DSCS for normal initial consonant /c/

In these two equations, $X_{H }$ and $X_{L}$ represent the HER and LER of the speech signal, respectively. $NX_{H}$ is obtained by the coordinate transformation of $X_{H }$;$NX_{L}$ is obtained by the coordinate transformation of $X_{L}$.${\text{Trans}}_{H }$ and ${\text{Trans}}_{L }$ are the amplitudes of translation of $X_{H }$ and $X_{L}$, respectively. The ${\text{Trans}}_{H }$ is the maximum value of HER, while the ${\text{Trans}}_{L }$ is the minimum value of LER. An example of DSCS is given in Fig. 12 based on normal initial consonant /c/.

As shown in Fig. 12, we can observe the amplitude variations of the signals in HER and LER in time domain. The proposed DSCS is useful for studying the relationship between the signals in HER and LER. In DSCS, since the translation influences only the amplitudes of signals in the HER and LER, their relation does not change in this translation process.

### The Pearson correlation coefficient calculation for the high-frequency region and low-frequency region of speech signal

**Fig. 13:**
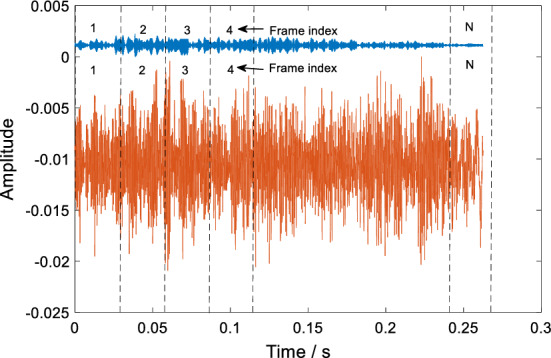
The framing process of pharyngeal fricative speech /c/. For each initial consonant, its LER and HER are correspondingly framed into N frames. The PCC value of each pair of frames is calculated. In this work, the N equals to 10. The frame size depends on the length of each initial consonant

The Pearson correlation coefficient (PCC) is used to calculate CSIFs in this work. PCC [62] can evaluate the relation between HER and LER of each initial consonant. The signals in HER and LER of each initial consonant have the same length in time domain. In the calculation process of PCC, the signals in high-frequency part and low-frequency part are initially framed. There is no overlap in adjacent speech frames. The framing process of pharyngeal fricative /c/ is shown in Fig. 13.

As shown in Fig. 13, the number (1, 2, 3…, *N*) is the frame index of the signals in HER and LER. The frames with the same frame index of HER and LER are as one group to calculate PCC. For the *n*th group of frames ($NX_{H} (n)$ and $NX_{L} (n)$), their PCC value *p*_{*n*} (*n *= 1, 2…, *N*, where *N* is the number of frames) is calculated using (5).

5 $$p_{n} = \frac{{E(NX_{H} (n)NX_{L} (n)) - E(NX_{H} (n))E(NX_{L} (n))}}{{\sqrt {E((NX_{H} (n))^{2} ) - E^{2} (NX_{H} (n))} \sqrt {E((NX_{L} (n))^{2} ) - E^{2} (NX_{L} (n))} }},$$

where $NX_{H} \left( n \right)$ is the *n*th frame of the HER, $NX_{L} \left( n \right)$ is the *n*th frame of the LER. *E*(*) is the expectation. Using (5), we calculate the PCC value of each pair frames. Then, each initial consonant is represented by these PCC values, which is called CSIFs.

### Proposed OSPP feature based on the prominent peak of the energy-concentration region

In the production process of speech, the places of articulation have great influences on the distribution of frequency components [63]. If the posterior displacement of the place of articulation occurs, the center frequency and the concentration degree of a prominent peak in the energy-concentration region decrease [30]. According to the places of articulation in normal production process of speech, the six types of initial consonants in this work can be classified as three types: apical (/c/, /s/ (IPA: [s])), blade-palatal (/ch/ (IPA: [ʈʂʰ]), /sh/), and alveopalatal sounds (/q/ (IPA: [tɕʰ]), /x/ (IPA: [ɕ])) [3]. These places of articulation are all in the oral cavity in the normal production process of speech, which differ from those in the production of pharyngeal fricative speech. Thus, there exist differences in the energy-concentration regions in the speech spectrum of pharyngeal fricative speech and normal speech.

For each type of initial consonant, there exist one or two energy-concentration regions in their speech spectrum [19, 30]. The results of studies [30, 64] demonstrate that the center frequencies of the six initial consonant types in normal speech are distributed in the lower half of the frequency range. Moreover, the changed place of articulation in pharyngeal fricative speech results in lower center frequencies than those in normal speech [64]. Then, one-third octave spectra are adopted in this research to emphasize the information in the low-frequency part of the speech signal [65]. In one-third octave spectra, frequency components ranging from 0 to 22,050 Hz are divided into 43 frequency bands [66]. These frequency bands in the lower frequency part are densely distributed with shorter bandwidths than those in the higher frequency part. This distribution of frequency bands is based on auditory properties that have excellent robustness to different speech signals [67].

**Fig. 14:**
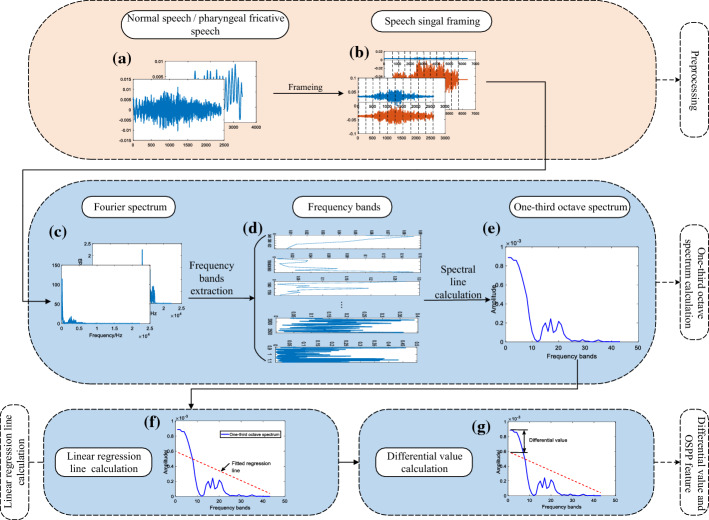
The block diagram of OSPP calculation. **a** Original speech signal; **b** speech signal framing without overlap; **c** calculated Fourier spectrum; **d** extracted frequency bands based on one-third octave spectrum; **e** calculated one-third octave spectrum; **f** linear regression fitting for one-third octave spectrum

Based on the above analysis on the relation between places of articulation and energy-concentration regions, a new feature is proposed in this subsection. Figure 14 illustrates the implementation procedure of the calculation process for the proposed feature. First, pharyngeal fricative speech and normal speech are segmented into *N* frames without overlap. Second, one-third octave spectra [68] are calculated based on the Fourier spectrum. Third, linear regression fitting is adopted in one-third octave spectra to normalize the amplitudes of frequency components. Finally, the OSPP feature is calculated based on the differential value from one-third octave spectra and the linear regression line of each frame.

The calculation process of the OSPP feature is described in detail as follows:

#### Preprocessing

Each initial consonant is initially segmented into frames. Then, the following calculation is carried out based on each frame.

#### One-third octave spectra calculation

The calculation of one-third octave spectra is divided into three steps:

1. Fourier spectrum calculation: the spectrum of each frame is calculated by Fourier transform (FFT size = 1024). It is performed frame by frame to calculate this spectrum.
2. Frequency bands extraction: according to one-third octave, the spectrum of each frame is divided into 43 frequency bands. There are three basic parameters in each frequency band: starting frequency, center frequency, and upper frequency. These three parameters are respectively represented by $f{}_{1}$, *f*_*c*_, and *f*_2_. The bandwidth of each frequency band equals (*f*_2_ − *f*_1_). The relationship of these three frequency parameters is described by (6), (7), and (8):
  6 $$f_{c} = \sqrt {f_{1} \times f_{2} }$$
  7 $$f_{2} /f_{1} = 2^{1/3}$$
  8 $$f_{c} /f_{1} = f_{2} /f_{c} = 2^{1/6}$$
3. Spectral line calculation: for each extracted frequency band of the 43 frequency bands, its spectral line ${\text{E}}\_\left\{ {\text{i}} \right\} (i = 1,{ 2} \ldots , 4 3$, where *i* is the frequency band index) is the mean value of the amplitudes in the frequency band. Then, the one-third octave spectra on the band level are represented by *E*.

#### Linear regression line calculation

The linear regression line is calculated by fitting the frequency band index of one-third octave spectrum to its corresponding *E*_*i*_ using the least square method [59]. Since the amplitudes of the spectrum used to calculate OSPP are affected not only by overall energy, but also by the window size of the spectrum analysis [68], linear regression fitting is adopted to normalize the overall amplitudes of one-third octave spectra.

#### Differential value and OSPP feature

The differential value is calculated from the most prominent peak value of one-third octave spectra and the corresponding value on the regression line that is directly below the peak. The OSPP feature of each initial consonant is formed by the group of each differential value. This calculation is described in (9).

9 $$DF\_\left\{ n \right\} \, = {\text{ peak}}\_\left\{ n \right\} - \, RL\_\left\{ n \right\},$$

where *DF* is the differential value of each frame, *n* (*n *= 1, 2…, *N*, where *N* is the number of frames) is the frame index, *peak* represents the maximum of the most prominent peak in one-third octave spectra, *RL* denotes the value of the linear regression line with the same horizontal ordinate of the prominent peak.

**Fig. 15:**
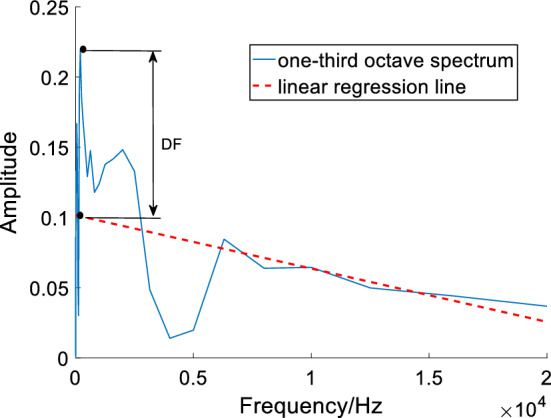
One-third octave spectrum and linear regression line of pharyngeal fricative /s/

An example of the one-third octave spectra and its linear regression fitting line of normal initial consonant /s/ is given in Fig. 15. The difference value is the distance between this double arrow.

## Data Availability

The data that support the findings of this study are available from [West China Hospital of Stomatologyl, Sichuan University] but restrictions apply to the availability of these data, which were used under license for the current study, and so are not publicly available. Data are however available from the authors upon reasonable request and with permission of [West China Hospital of Stomatologyl, Sichuan University].

## Abbreviations

AUC: Area under the receiver operating characteristic curve
OSPP: Octave spectrum prominent peak
CSIFs: Correlation of signals in independent frequency bands
EMAs: Amplitudes of eight one-third octave bands
FMAs: Amplitudes of four one-third octave bands
PCC: Pearson correlation coefficient
DSCS: Double signal coordinate system
LER: Low-energy region
HER: High-energy region
ICPD: Independent consonant prominent distribution
PP: Pitch period
VTA: Vocal tract area
VTG: Vocal tract gain

## References

[1] He L, Zhang J, Liu Q, Yin H, Lech M (2014). Automatic evaluation of hypernasality and consonant misarticulation in cleft palate speech. IEEE Signal Process..

[2] Kosowski TR, Weathers WM, Wolfswinkel EM, Ridgway EB (2012). Cleft palate. Semin Plast Surg.

[3] Lei L (2004). Speech therapy for cleft palate.

[4] Trost-Cardamone J, Bzoch R, Kenneth R (1997). Diagnosis of specific cleft palate speech error patterns for planning therapy or physical management needs. EdCommunicative disorders related to cleft lip and palate.

[5] Hermes Z, Barlaz M, Shosted R, Liang ZP, Sutton B. Phonetic correlates of pharyngeal and pharyngealized consonants in Saudi, Lebanese, and Jordanian Arabic: An rt-MRI Study. 201–205. In: proceedinga 3rd annual international conference INTERSPEECH., Sweden, 2016. pp. 201–205.

[6] Oren L, Kummer A, Boyce S (2020). Understanding nasal emission during speech production: a review of types, terminology, and causalit. Cleft Palate Craniofac J.

[7] Khattab G, Al-Tamim J, Alsiraih W (2018). Nasalisation in the production of Iraqi Arabic pharyngeals. Phonetica..

[8] Park YH, Jo HJ, Hong IS, Leem DH, Baek JA, Ko SO (2019). Treatment of velopharyngeal insufficiency in a patient with a submucous cleft palate using a speech aid: the more treatment options, the better the treatment results. Maxillofac Plast Reconstruct Surg.

[9] Vijayalakshmi P, Reddy MR, O’Shaughnessy D (2007). Acoustic analysis and detection of hypernasality using a group delay function. IEEE Trans Biomed Eng.

[10] Hong BY, Liu WL, Li F, Gao ML. Study on test methods for main optical characteristics of medical endoscopes. In: proceeings of 3th Annual IMCCC, Beijing, 2013. pp. 190–194.

[11] Ara P, Cheng S, Heimlich M, Dutkiewicz E. Investigation of in-body path loss in different human subjects for localization of capsule endoscope. In proceedings 37th Annual IEEE Eng Medical Biological Society. 2015. pp. 5461–4. 10.1109/EMBC.2015.7319627 26737527

[12] . Xue PY. Analysis and Recognition of Pathological Speech in Patients with Dysarthria. Ph. D. theses, Dept. Electro. Sci. and Tech., Taiyuan Univ. of Tech., Taiyuan, Shanxi, China. 2019.

[13] Garcia AF, Castro Marino VC, Pegoraro-Krook MI, Guerra TA, Rillo Dutka JC (2014). Nasalance during use of pharyngeal and glottal place of production. CoDAS..

[14] Guerra TA, Marino VCC, Rocha DCD, Jaco MF, Pegoraro-Krook MI, Dutka JCR (2016). Nasalância na presença e ausência da fricativa faríngea. Revista CEFAC..

[15] Eliküçük CD, Kayıkc MEK, Aydınl FE, Çalış M, Özgür FF, Öztürk M, Günaydın RÖ (2017). Investigation of the speech results of posterior pharyngeal wall augmentation with fat grafting for treatment of velopharyngeal insufficiency. J Cranio-Maxillofac Surg.

[16] Raul HM, Kenneth S, Kristen HSOBR (2019). Effect of prompts for restructuring oral muscular phonetic targets (PROMPT) on compensatory articulation in children with cleft palate/lip. Global Pediatric Health..

[17] Godino-Llorente JI, Gomez-Vilda P, Blanco-Velasco M (2006). Dimensionality reduction of a pathological voice quality assessment system based on gaussian mixture models and short-term cepstral parameters. IEEE Trans Biomed Eng.

[18] Zhang HY, Zhou Y, Yu J, Wang D, Zhang CH (2015). Spectrum analysis system for computer application in professional voice assessment. J Harbin Med Univ.

[19] Zhu YS, Liang JL (2001). Phonetic characteristics of cleft palate and its influencing factors. Chin J Plast Surg.

[20] Wang GM, Pan WY (2000). Clinical application and evaluation in analysis of articulation disorders with CSL. Chin J Oral Maxillofac Surg.

[21] Nikhila K, Prasad H (2017). A study on patterns of compensatory articulation errors with reference to age of surgery in children with repaired cleft lip and palate. Global J Otolaryngol.

[22] Segura-Hernández M, Valadez-Jiménez V, Ysunza P, Sánchez-Valerio A, Arch-Tirado E, Lino-González A (2019). Acoustic analysis of voice in children with cleft lip and palate following vocal rehabilitation Preliminary report. Int J ric Otorhinolaryngol..

[23] Xiao Y, Liang MG. Automatic detection of pharyngeal fricatives in cleft palate speech. In: proceedings of 4th Annual, international conference institute of information science, Beijing. 2015. pp. 591–7.

[24] Xiao Y. acoustic analysis of compensatory articulation in cleft palate speech. M.S. thesis, Dept. Electron. Eng., Beijing Jiaotong Univ., Beijing, China. 2016.

[25] He Fei, Zhou Geyi, He Xinyi, Yin Heng, He Ling (2018). Automatic detection of pharyngeal fricative in cleft palate speech. MATEC Web of Conferences.

[26] Fu J, Mo X, HUANG S R, MENG Y X, Yin H, He L. Automatic detection algorithm of pharyngeal fricatives in cleft palate speech based on LPIF and feature selection. DEStech transactions on engineering and technology research. Xiamen, China. 2018. pp. 359–63.

[27] Hansen JHL, Gavidia-Ceballos L, Kaiser JF (1998). A nonlinear operator-based speech feature analysis method with application to vocal fold pathology assessment. IEEE Trans Biomed Eng.

[28] Asaei A, Cernak M, Bourlard H (2017). Perceptual information loss due to impaired speech production. IEEE/ACM Trans..

[29] Vargas J, McLaughlin S (2011). Speech analysis and synthesis based on dynamic modes. IEEE Trans..

[30] Bao HC (2014). Summary of experimental phonetics. Enlarged edition.

[31] Orozco-Arroyave JR, Belalcazar-Bolanos EA, Arias-Londono JD, Vargas-Bonilla JF, Skodda S, Rusz J (2015). Characterization methods for the detection of multiple voice disorders: neurological, functional, and laryngeal diseases. IEEE J Biomed Health Inf.

[32] Huang F, Xie G, Xiao R. Research on ensemble learning. In: Proceeding of annual international conference artificial intelligence and computational intelligence. Fu Zhou, China, 2009. pp. 249–52

[33] Singh N, Rao S. Online ensemble learning approach for server workload prediction in large datacenters. In: Proceedings of 11th annual international conference machine learning and applications. Florida, USA. 2012. pp. 68–71.

[34] He Y, Wang J, Qin LX, Mei L, Shang YF, Wang WF. Clustering algorithm based on ensemble learning, ICSSC. 2013. pp. 300–5

[35] Su L, Liao HZ, Yu ZT, Zhao Q. Ensemble learning for question classification. In: Proceeding of IEEE international conference intelligent computing and intelligent systems. Shanghai, China. 2009. pp. 501–5.

[36] Shaikhinaa T, Lowe D, Daga S, Briggs D, Higgins R, Khovanova N (2019). Decision tree and random forest models for outcome prediction in antibody incompatible kidney transplantation. Biomed Signal Process Control.

[37] Duysak H, Yigit E (2020). Machine learning based quantity measurement method for grain silos. Measurement.

[38] Wong TT (2015). Performance evaluation of classification algorithms by k-fold and leave-one-out cross validation. Pattern Recogn.

[39] Zarei S, Yosefvand F, Shabanlou S (2020). Discharge coefficient of side weirs on converging channels using extreme learning machine modeling method. Measurement.

[40] Fan JL, Yue WJ, Wu LF, Zhang FC, Cai HJ, Wang KJ, Lu XG, Xiang YZ (2018). Evaluation of SVM, ELM and four tree-based ensemble models for predicting daily reference evapotranspiration using limited meteorological data in different climates of China. Agric For Meteorol.

[41] Bergmeir C, Hyndman R, Koo B (2018). A note on the validity of cross-validation for evaluating autoregressive time series prediction. Comput Stat Data Anal.

[42] Patil K, Nagwani NK, Tripathi S. A parametric study of partitioning and density based clustering techniques for Boxplot generation. In: Proceedings of 3th annual international conference convergence in technology. Pune, India. 2018, pp. 1–5.

[43] Williamson DF, Parker RA, Kendrick JS (1989). The box plot: a simple visual method to interpret data. Ann Intern Med.

[44] Yin H, Guo CL, Shi B, Zhao SF (2013). A preliminary study on the consonant articulation of older patients with cleft palate. West China J Stomatol.

[45] Min ZY, Li F, Zhang YY, Hu MF (2018). A study on the consonants characteristics of articulation disorders adults with repaired cleft palate. J Audiol Speech Pathol.

[46] Zhao WH, Huang NE (2004). A study of the characteristics of white noise using the empirical mode decomposition method. Proceedings of the Royal Society of London..

[47] Zhou XY, Wu YS (2002). Study of similarities for fluid-dynamic noise. ACTA ACUSTICA..

[48] Ke L, Lin YK, Zeng Z, Zhang LF, Meng LK (2018). Adaptive change detection with significance test. IEEE Access..

[49] Li P, Zhang BS, Weng Y, Rajagopal R (2017). A sparse linear model and significance test for individual consumption prediction. IEEE Trans Power Syst.

[50] Guerriero M, Pozdnyakov V, Pozdnyakov J, Willett P (2010). A repeated significance test with applications to sequential detection in sensor networks. IEEE Trans Signal Process.

[51] Heinrich SP (2009). Permutation-Based Significance Tests For Multiharmonic Steady-State Evoked Potentials. IEEE Trans Biomed Eng.

[52] Sáenz-Lechón N, Godino-Llorente JI, Osma-Ruiz V, Gómez-Vilda P (2006). Methodological issues in the development of automatic systems for voice pathology detection. Biomed Signal Process Control..

[53] Brzezinski D, Stefanowski J, Prequential AUC (2017). Properties of the area under the ROC curve for data streams with concept drift. Knowl Inf Syst.

[54] He L, Zhang J, Liu Q, Zhang JP, Yin H, Margaret L (2018). Automatic detection of glottal stop in cleft palate speech. Biomed Signal Process Control.

[55] Proctor MI, Shadle CH (2010). Iskarous k, Pharyngeal articulation in the production of voiced and voiceless fricatives. J Acoust Soc Am.

[56] Zhu YS, Wu WH, Yan S, He BH, Shi JJ (2001). Analysing misarticulation of post-operation cleft palate speech applying acoustic technology. J Clin Stomatol.

[57] Trost J (1981). Articulatory additions to the classical description of the speech of persons with cleft alate. Cleft Palate J..

[58] Kruskal WH, Wallis WA (1952). Use of ranks in one-criterion variance analysis. J Am Stat Assoc.

[59] Mohammed GA, Hou M (2016). Optimization of active muscle force-length models using least squares curve fitting. IEEE Trans Biomed Eng.

[60] Xue JH, Titterington DM (2011). t-Tests, F-tests and otsu’s methods for image thresholding. IEEE Trans Image Process.

[61] Shang Y. Research on Parallel Filtering Algorithms and Systolic Structure. Ph. D. theses, Dept. Electro. Sci. and Tech., Xidian. Univ., Xi’an, Shanxi, China. 2000.

[62] Chen JB, Huang YT (2008). On the importance of the pearson correlation coefficient in noise reduction. IEEE Trans..

[63] Gautam S, Singh L. Developmental changes of spectral parameter in children speech. In: Proceedings of 3th annual international conference signal processing and integrated networks, Noida. 2016, pp. 220–5.

[64] Li SP, Tao WT (2016). Acoustic characteristics of Mandarin affricates. J Tsinghua Univ..

[65] Kataoka R, Warre DW, Zajac DJ, Mayo R, Lutz RW (2001). The relationship between spectral characteristics and perceived hypernasality in children. J Acous Soc Am.

[66] Zhou HF, Lopez-Arteaga I, Nijmeijer H (2016). Broadband planar nearfield acoustic holography based on one-third-octave band analysis. Appl Acoust.

[67] Vogel AP, Ibrahim HM, Reilly S, Kilpatrick N (2009). A comparative study of two acoustic measures of hypernasality. Speech Lang Hear Res..

[68] Hillenbrand J, Cleveland RA, Erickson RL (1994). Acoustic correlates of breathy vocal quality. J Speech Lang Hear Res..

